# The Comorbidity of Violence Exposure, Mental Health, Cognitive Functioning, and Disabilities in Incarcerated Youth

**DOI:** 10.1007/s40653-026-00960-w

**Published:** 2026-08-26

**Authors:** Suzanne Perkins, Sriamsha Dubbaka, Erin Wang, Michaela Wu, Wendy Su, Vanessa Assaf, Julianne Park, Joanne Smith-Darden, Sandra Graham-Bermann

**Affiliations:** 1 Institute for Social Research, University of Michigan–Ann Arbor, Ann Arbor, United States; 2 Lurie Children’s Hospital, Chicago, United States; 3 Department of Psychology, University of Michigan–Ann Arbor, Ann Arbor, United States; 4 School of Social Work, Michigan State University, East Lansing, United States

**Keywords:** Violent offenders, Juvenile Justice, Comorbid Diagnosis, Executive Functioning, Adverse Childhood Experiences

## Abstract

Objective. The goal of the present study was to ascertain the prevalence rates of violence exposure (VE) and disabilities in a population of incarcerated male adolescents using records searches, survey research, and individual academic and cognitive testing. Methods. A sample of 115 incarcerated male youth completed self-reported measures of VE, including adverse childhood experiences (ACE), child abuse and neglect, interpersonal violence experiences (IPV), and community violence exposure (CV), including current functioning and individual testing of cognition and disability diagnosis (evaluating both features of cognitive and mental health). Results. 90% of participants had experienced at least mild physical abuse, two-thirds reported high ACEs, and one-third reported severe prior sexual abuse (SA) using the CTQ. Over 60% had a prior disability in their records, and over 75% were diagnosable during individual cognitive testing. Just over 50% of youth fit criteria for a mental health diagnosis, and with cognitive disabilities, this prevalence rate increased to 85% of the population. Youth with disabilities had higher rates of VE. Conclusion. Few existing studies analyze combined data related to prior VE, disability diagnosis using individual measures, while testing for current cognitive functioning in incarcerated youth. The present study determined that high rates of disability among incarcerated youth were largely driven by cognitive and academic disabilities, rather than mental health problems, indicating a pervasive problem with cognitive disabilities in this population. Cognitive disabilities, particularly in youth with prior histories of VE, are an intervention point that has the potential to reduce recidivism.

**Supplementary Information:**

The online version contains supplementary material available at https://doi.org/10.1007/s40653-026-00960-w.

## Introduction

Incarcerated youth are a vulnerable population with complex developmental and health backgrounds including high rates of exposure to violence of all kinds, particularly interpersonal family violence including child abuse (Baglivio et al. 2014; Brannon et al. 1989; Gore-felton et al. 2002; Haapasalo and Moilanen 2004; Huang et al. 2006; Jones et al. 2021a, b; Lewis et al. 1979a; Papalia et al. 2022), behavioral and mental health concerns (Domalanta et al., 2003; Gaete et al., 2018; Gore-felton et al., 2002; Heller et al., 2022; Papalia et al., 2022; Teplin et al., 2002), cognitive functioning deficits (Mallett et al., 2023; Papalia et al., 2022; Whittingham et al., 2020), and high rates of disability (Li et al., 2023; Whittingham et al., 2020). Neuroscience studies of youth with violence exposure backgrounds (VE) show that VE and other ACEs disrupt the developmental processes of fear and cognitive control processing through the experiences of threat and deprivation (Johnson et al., 2021; McLaughlin & Gabard-Durnam, 2022; Sheridan et al., 2022).

Fear and cognitive control processing are largely dependent on prefrontal cortical top-down control of responses (Sheridan et al., 2017; Weissman et al., 2019), and deficits in top-down control are the basis for behavioral deficits in executive functioning (Rueda et al., 2005).

Studies have demonstrated that childhood VE is associated with changes in brain functioning during emotion (Hein & Monk, 2016) and cognitive function (S. C. Perkins et al., n.d.), suggesting that VE in childhood has a unique developmental influence on both mental health and long-term cognitive outcomes. This indicates that both mental health and cognitive outcomes may be the result of brain-based cognitive responses that have developed in the context of trauma and stress in this population. Understanding VE -to -disability mechanisms in this population has the potential to drive targeted interventions that incorporate trauma-informed care with academic and special needs programming that may help reduce recidivism and increase post-incarceration outcomes. A first step in developing a mechanistic theory of VE -to -disability is to understand the prevalence of both VE and disability in incarcerated youth.

### Interpersonal Violence Exposure in Incarcerated Youth

VE is a national problem that is particularly salient for incarcerated youth. The Crime Against Children Research Center found that 4 in 10 youth (41.2%) from a nationally representative sample had experienced at least one incident of physical assault in the past year, approximately 40% had witnessed violence at some point in their lives in the United States (Finkelhor et al., 2025), and 38% of children reported the prevalence of one ACE (Ding et al., 2025). Other studies of incarcerated youth and adults also show higher prevalence of VE and other ACEs (Baglivio et al., 2014, 2015, 2020; Fox et al., 2015; Wolff et al., 2017) than the general population (Finkelhor et al., 2025).

While VE is a common ACE among incarcerated and court-involved youth, it only represents one part of the broader ACEs also common in this population. Incarcerated youth are four times more likely to present with four or more ACEs than the non-juvenile population (Baglivio et al., 2014, 2015) and this exposure is more likely to lead to an increased risk of violent acts and violent recidivism (Baglivio et al., 2020). Moore (2013) found that one third of incarcerated youth had a parent with substance use disorder and nearly half had a parent formerly incarcerated. Parental history of incarceration is a significant predictor of later criminal behavior along with CM ACEs (Casey et al., 2025) and serious violent offenders appear to be more likely to experience every type of ACE (Kimonis, 2011; Fox et al., 2015), suggesting that exposure to ACEs are a critical factor in the development of disorders of control, including both mental health and cognitive control.

### Behavioral and Mental Health in Incarcerated Youth

Epidemiological studies have constantly reported high rates of psychiatric disorders, mental health outcomes, and developmental challenges among incarcerated youth, suggesting that juvenile youth experience multiple complex forms of vulnerability (Baglivio et al., 2020; Heller et al., 2022). Prevalence rates of mental health disorders in incarcerated youth often exceed two thirds of populations (Beaudry et al., 2021; Teplin et al., 2002). VE is a significant risk factor for poor mental health outcomes and engagement in risky behaviors (Annett et al., 2023; Skarupski, 2016) such as substance use, violent behavior, and attitudes towards aggression, including increased support for aggressive attitudes, self-reported aggression, and lifetime violent offending (Ball, 2005a; Moore et al., 2013). These conditions frequently occur with violence exposure, suggesting that there might be many forms of vulnerability that incarcerated youth experience.

### Cognitive Functioning in Incarcerated Youth

An early study connected lower cognitive performance with violence in offenders (Lewis et al. 1979b). More recent work has demonstrated lower language processing (Chow et al., 2022), fluid intelligence (Herrero et al., 2010) and traumatic brain injury (TBI, Davies et al., 2012) as risk factors for cognitive processing deficits.

### Disability in Incarcerated Youth

One often overlooked factor associated with youth incarceration is high rates of disabilities (Mallett et al., 2023). A recent Canadian study found that disabilities were more than double in the adult incarcerated population (Whittingham et al., 2020). One study found that of the 66% of inmates reported having a disability, 40.4% had non-psychiatric disabilities, which consisted of cognitive disabilities, ADHD, or enrollment in “special education”, indicating this as an important area to investigate (Bixby et al., 2022). A recent American study found that around 20% of justice involved youth have behavioral special needs and around 12% have learning needs (Hayes et al., 2025). A review of intellectual disabilities found higher rates worldwide in incarcerated individuals than in the general population (Fazel et al., 2008).

Like VE, disabilities may be a risk factor for criminal offending: Mallett and colleagues (2023) found that children who have received services for learning disabilities have an increased risk of crime and delinquency, and later incarceration. Most studies of disabilities in incarcerated populations rely on records searches or self-reports which may inaccurately estimate rather than use diagnostic tools to assess cognitive and academic functioning (Chow et al., 2022; Fazel et al., 2008; Young et al., 2015). In fact, meta-analytic and systematic reviews on disabilities are limited because researchers have not adequately characterized multiple disabilities and assessed current cognitive function in incarcerated populations, particularly using diagnostic testing. Finally, disability is likely a risk factor for VE in this population (Hayes et al., 2025). One goal of the present study is a comprehensive assessment of youth using both medical and educational records review, and multiple diagnostic and cognitive assessments.

### Study Objectives

To understand how high rates of VE, mental health diagnosis, and cognitive and academic disabilities may interact to lead to violent offending, it is important to understand comorbidity in juvenile justice populations. The goal of the present study is to ascertain prevalence rates of VE and both physical and mental health disabilities in a population of incarcerated male adolescents using records searches, survey research, and individual academic and cognitive testing. To develop targeted interventions for this population, it will be necessary to understand the basic mechanisms of cognitive functioning and VE as a mechanism for intervention.

## Methods

### Participants

Participants were recruited from an all-male youth incarceration facility in southeastern XXX and included all willing participants. Human Subjects approval was granted by the University of XXX Institutional Review Board in consultation with the State Office of Human Services, which approved the facility’s cooperation. A federal certificate of confidentiality was obtained from the National Institutes of Health to protect participant data. Further details on recruitment and consent procedures have been previously reported (Author reference, 2011; Author reference, 2012). Data from the study are publicly available through (Author et al., 2023).

### Record Review

The review of medical, educational, and intake records was carried out by six trained graduate and undergraduate students. The records were coded for medical and educational diagnoses and criminal offenses. More information on the collection of each of these records can be found in the supplemental (see Supplemental Text 1 and Perkins et al., 2024).

### Survey Methods

Participants completed a survey booklet and two validated self-report measures of functioning. The booklet included demographic measures, family and educational history, and 11 validated scales. The validated scales included measures of violence exposure, delinquency, prior offending, family functioning, and mental health functioning.

#### Violence Exposure

Violence exposure was assessed with four well-known measures of children’s exposure to violence in the domains of ACE, interpersonal violence (IPV) in the family, childhood experiences of trauma, and community violence exposure (CVE).

#### Adverse Childhood Experiences (ACE)

13 ACE items included criminality in the family, parental use and trafficking in alcohol/drugs, childhood neglect, physical and sexual abuse, interpersonal violence in the family, and poverty. A total score was obtained.

#### Interpersonal Violence (IPV)

IPV was assessed with an 18-item modified version of the Conflict Tactics Scale (CTS-modified, Straus, 1979). Participants were asked to rate the frequency of experiencing the four types of maltreatment on a five-point scale (1 = never, 5 = every day). The version used in this study was modified to include violence experienced by the youth from siblings and to differentiate between experiences from the ages of 6–12 and the ages of 13–18. Four sub-scale means were calculated for Personal Violence, Sibling Violence, Parental Violence, and Interpersonal Violence. The Personal Violence sub-scale evaluates the individual’s report of their own violence. The internal reliability of each of the four types of relationships was excellent, with Cronbach’s alpha ranging from 0.93 to 0.97.

#### Childhood Trauma Questionnaire

A modified 34-item Childhood Trauma Questionnaire (Bernstein et al., 1994) was administered to measure the experience of subtypes of childhood abuse and neglect on a five-point Likert scale (1 = never true, 2 = rarely true, 3 = sometimes true, 4 = often true, 5 = very often true). Recognizing that youth may often misinterpret sexual abuse behaviors in unhealthy relationships as “dating” and expressions of affection, an additional question was added to the sexual abuse sub-scale: “I had sex with an adult or with someone a lot older than me (someone at least 5 years older than me).” Sub-scales were created of emotional abuse and neglect, physical abuse and neglect, and sexual abuse. Due to the modification of the scale from the 28-item CTQ, mean scores were used for low, moderate, and severe classification.

#### Community Violence Exposure (CVE)

A modified Richters and Martinez (Richters & Martinez, 1993) Things I have Seen and Heard sixteen-item scale was used to assess CVE. The items were on a Likert scale from never to many times (0–3) and were modified to add a single item querying exposure to seeing people having sex, based on previous work with this population (Burton et al., 2011). A total score and sub-scales for experiencing and witnessing were created.

#### Behavioral and Mental Health

##### Behavioral Health

Participants completed four self-report behavioral health functioning measures that included delinquency, self-esteem, and two measures of attachment style.

##### Self-Reported Delinquency

Delinquency was measured with a 38-item version of the Self-Reported Delinquency Scales from the Denver Youth Study Crime and Delinquency protocol (SRD; Huizinga et al., 1991). Items assessed Delinquency based on the participants’ self-reported descriptions of their delinquent activities, whether he/she ever committed an act, how many times in the past year, if others were involved, and if he/she was under the influence of alcohol or drugs while committing it, for each type of delinquent act. Sub-scales of public disorder, theft, violence, and financial crimes were created. Cronbach’s alphas of the 37-item modified version of the Denver Youth Study SRD ranged from 0.64 − 0.92.

Self-esteem, attachment style, and relationship were also assessed using the Coopersmith self-esteem inventory, relationship questionnaire, and relationship scales questionnaire respectively (see Supplemental Text 2).

##### Mental Health

Participants completed three validated mental health self-report measures that provide clinical diagnostic coding for youth mental health, depression, and posttraumatic stress disorder.

##### Youth Self Report

The Youth Self Report (Achenbach & Ruffle, 2000) is a self-report measure for youth ages 11 to 18. The 112-item assessment is on a three-point scale (0 = none, 1 = somewhat true, 2 = very often true). The reliability alpha in this study is between 0.7 and 0.93.

##### Beck Depression Inventory

The Beck Depression Inventory self-report was used to determine the severity of depression symptoms in youth (Beck, 1967). The measure consists of 21 items on a four-point Likert scale. A total score was calculated, to score for minimal, mild, moderate, and severe depression (Palmer & Binks, 2008). Reliability in the current study yielded a Cronbach’s alpha of 0.4 to 0.85.

##### Trauma Symptom Checklist

The 42-item Trauma Symptom Checklist for Children (TSCC-A; Briere, 1996) was used to measure the prevalence of Post Traumatic Stress Disorder symptoms on a 4-point scale from 0 (never) to 3 (almost all the time). Two items assessing suicidality were removed for this study at the request of reviewers. Five sub-scales were created for post-traumatic stress symptoms (PTSS), anxiety, dissociation, anger, and depression. Clinical level of traumatic stress symptoms was determined based on subscale total scores (0–15 = mild, 16–30 = moderate, and 31 + = severe). The internal reliability of each sub-scale ranged from 0.72 to 0.85.

### Executive Function and Cognitive Assessment

Participants completed four computerized cognitive assessments of executive function and were individually evaluated on measures of current academic and intellectual functioning.

#### Executive Function

The computerized battery included five executive functioning tasks: Go – No Go, Anti-Saccade, Task Switching, Flanker Shape, and Shape Matching. For each measure, standard assessment of performance was calculated. The Go-No Go (GNG) task is a stop-signal response inhibition task with repeated button presses until participants receive a prompt to disinhibit that behavior (Nigg, 2000). The Anti-Saccade (AS); (Friedman & Robbins, 2022) is a visual processing response inhibition task that requires oculomotor inhibition. Task switching (TS) is a set switching task that requires the subject to switch mental sets between two different tasks (Friedman & Miyake, 2004; Rogers & Monsell, 1995). The Flanker Shape (FS) task requires interference processing, specifically resistance to distractor interference (Friedman & Miyake, 2004). Shape Matching (SM) also requires distractor interference, but it required matching irregular shapes as well (Friedman & Miyake, 2004). Additional information about each of these tests can be found in the supplemental (see Supplemental Text 3).

A poor performance score was calculated, and a count of poor performance indicators generated. These poor performance indicators were then used to assess task performance in context and compare to disability diagnosis.

#### Cognitive Assessments

##### Kaufman Brief Intelligence (KBIT-2)

The Kaufman Brief Intelligence Test Second Edition was used to evaluate verbal and non-verbal intelligence in participants (Kaufman, 1990, 1993).

##### Wide Range Achievement Test (WRAT-3)

The Wide Range Achievement Test 3 was used to evaluate participants’ academic skills in reading, mathematics, and spelling (Wilkinson, 1993).

##### Wisconsin Card Sort Task (WCST-CV)

Processing problems related to frontal lobe dysfunction, processing problems related to frontal lobe lesions, and learning disabilities were assessed using the Wisconsin Card Sort Computerized Version 4 (WCST-CV4; (Heaton, 1993).

##### Behavior Rating Scale of Executive Function (BRIEF-SR)

The 86-item Behavior Rating Inventory of Executive Function Self-Report Scale (Guy et al., 2004) was used to evaluate self-reported executive functioning, including: Inhibit, Shift, Emotional Control, Monitor, Working Memory, Plan/Organize, Organization of Materials, and Task Completion. Sub-scales were combined into three indices according to the manual. Raw scores were converted to T- scores and percentiles.

### Disability Diagnosis

Using eight academic and three mental health measures, where clinical diagnosis is possible, an overall number of super-clinical scores was calculated for each youth by a certified special educator with two master’s degrees, one in education and one in psychology.

## Results

### Participants

The sample consisted of a convenience sample of 115 youth from a 200-bed facility where the daily census fluctuated but did not generally rise above 180. The 115 participants returned a signed consent form, signed an assent form, and were available for testing when researchers were present. There were 123 total youth who both consented and assented; however, some were not available for testing due to release from the facility, appointments, or behavioral restrictions, resulting in the final sample of 115. Missing data was common due to similar challenges and was not imputed. Scale *N’*s are reported in tables.

### Record Review

From the medical records, the highest counts of medical diagnoses were depression, asthma, antipsychotics, and ADHD. Around half of the incarcerated youth were on medications associated with depression. The criminal records indicated that the largest part of the sample was Class 2 and Class 3 sexual offenders (see Supplemental Text 4).

#### Educational Records

Over 60% of youth had previously participated in an Individualized Education Program (IEP), and there was evidence that over 62% had disability diagnoses (see Supplemental Table 2). The most prevalent special education disabilities in youth were ADHD (41.5%), emotional disturbance (40.7%), and vision (47.2%). Other notable disabilities include cognitive disabilities (9.8%), speech (10.6%), communication (8.1%), and hearing (8.1%).

### Survey Methods

#### Demographics

The sample consisted of 115 youth ranging from 13 to 20 years old, between 7th-grade and first year of college education (Author reference et al., 2011). Almost a third of the youth came from a two-parent family of origin, with close to 45% from single-parent families; most were mother-headed households (see Supplemental Table 44). The sample was fairly evenly divided between races, with approximately one-third in white, black, and multiracial self-identification.

Nearly 90% of individuals self-reported previous locked detention, over a third reported past placement in foster care, and nearly 20% reported previous residence in a group home. Previous psychological (85.3%) and substance abuse treatment (15.0%) were also common (see Supplemental Table 55).

#### Violence Exposure

Youth were asked whether they believed they had been sexually, physically, or emotionally abused, which resulted in over 60% reporting abuse in each category sexual abuse (SA) *n* = 69 (60.5%), physical abuse (PA) *n* = 72 (63.2%), emotional abuse (EA) *n* = 84 (73.7%). Just over 45% of youth reported that they had experienced SA, PA, and EA altogether, while 81.7% reported at least one type of abuse.

#### Adverse Childhood Experiences

ACEs’ total scores ranged from 0 to 12, out of 13 possible items, with a mean score of 4.65 (see Table 1). There were five youth (4.8%) who reported no ACEs, 30 (28.6%) who reported between one and three ACEs, and 70 youth who reported four or more ACEs (66.7%).

**Table 1:** Descriptive Statistics for Violence Exposure

		*N*	Min					Max	Mean			SD				Alpha
Adverse Childhood Experiences (ACEs)																
ACEs total score		105	0					12	4.65			2.74				0.68
Conflict Tactics Scale (CTS-modified)																
Childhood maltreatment		97	1					5	1.89			0.08				0.94
Witnessing IPV		97	1					5	1.64			0.08				0.94
Sibling Violence		101	1					4.47	1.95			0.07				0.93
Personal Violence		100	1					3.81	1.97			0.07				0.93
Childhood Trauma Questionnaire (CTQ-modified)																
Emotional Neglect		103	1					5	2.69			1.14				0.92
None = 60 (58.8%)		Low = 9 (8.8%)						Moderate = 5 (4.9%)								
Emotional Abuse		102	1					5	2.40			1.17				0.88
None = 57 (55.9%)		Low = 14 (13.7%)						Moderate = 6 (5.9%)				Severe = 28 (27.5%)				
Physical Neglect		103	1					4.67	2.19			0.74				0.75
None = 41 (41.4%)		Low = 25 (25.3%)						Moderate = 6 (4.9%)				Severe = 2 (27.3%)				
Physical Abuse		102	1					5	2.41			0.87				0.87
None = 39 (38.2%)		Low = 18 (17.6%)						Moderate = 3 (2.9%)				Severe = 42 (41.2%)				
Sexual Abuse		103	1					5	2.26			1.17				0.86
None = 20 (29.4%)	Low = 3 (33.3%)							Moderate = 4 (3.9%)				Severe = 34 (33.3%)				
Community Violence (CV)																
CV Experience		101	0					3	1.35			0.72				0.71
CV Witnessed		101	0					3	1.11			0.73				0.90
Total Score		101	0					2.93	1.18			0.66				0.89

#### Conflict Tactics

The CTS demonstrated a high frequency of overall IPV experienced and committed by the participants across childhood (M = 68.42, SD = 2.55). Each of the four subcategories of violence ranged from a score of 1 (no violence) to 5 (severe violence). The average violence score was approximately two in each category, corresponding to mild violence exposure.

#### Childhood Trauma Questionnaire

Scores on the CTQ ranged from 1 to 5 in each subtype of childhood trauma (see Table 1). The overall mean scores were at least low on emotional abuse (EA), physical neglect (PN), physical abuse (PA), and sexual abuse (SA), with a mean PA score of moderate level. Three or more severe traumatic exposures were reported by 28 youth (22%) of the population and over 50% reported severe traumatic exposure on at least one subcategory of childhood trauma (*n* = 67). Between 25 and 41% of the population qualified for severe trauma on each of the five subtypes. At least 25% of the sample met criteria for Severe in all five subtypes of abuse. According to the cut-off scores, our data indicated that only 26% of the population reported no maltreatment in any of the five categories, while 12 youth (9.8%) reported at least a low level of maltreatment on one scale, 21–22 reported low or greater maltreatment on two, three, or four scales, and 15 youth reported at least a low level of maltreatment on all five scales. The rate of PA, SA, severe ACEs, and severe traumatic exposure were combined to examine the prevalence of severe violence exposure in the population. Using these three measures 15%, 31%, and 56% reported a severe level of violence exposure on one, two, or three of these measures.

#### Community Violence Exposure

The average score on the Things I Have Heard and Seen Community Violence measure was 1.18, which is just over a mean of “once or twice” on a 0–3 scale with approximately the same mean for experienced and witnessed sub-scales (see Table 1). Both sub-scales and the total score produced high Cronbach’s alpha scores.

#### Behavioral Health

Mean endorsement of prior delinquency on the SRD ranged from 31 times (violence) to 70 times (theft) in the last year (see Supplemental Table 6). The self-esteem, attachment style, and relationship scale questionnaire results can be found in the supplemental section (see Supplemental Table 6).

#### Mental Health

Mental health subscale descriptives can be found in Supplemental Table 6.

#### Youth Self Report (YSR)

Means of all eight sub-scales of the Youth Self-Report were between 0 and 1, representing between “Not true” and “Somewhat or Sometimes True.” The presence of clinical levels of mental health problems is high for all eight sub-scales of the YSR. Between 12.2 and 45.2% of youth scored above the cut-off for borderline mental health on each of the eight sub-scales. Over 40% of the population had a score above the clinical level on at least one sub-scale, with nearly 8% scoring at the super-clinical level on four or more sub-scales.

#### Beck Depression Inventory (BDI)

Means for the three sub-scales from the Beck Depression Inventory (BDI-II), General Depression (GD), Performance Impairment (PI), and Somatic Complaints (SC), were less than 1 on the 0–3 Likert scale. Over 30% of incarcerated youth reported at least mild depression, and 17% of the youth had moderate depression symptoms.

#### Trauma Symptom Checklist for Children (TSCC)

Mean T-scores on the five sub-scales ranged from approximately 33 to over 80 when the clinical cut-off is a t-score of 65. Just over 20% of the sample was above clinical on at least one sub-scale of the TSCC.

### Executive Function and Cognitive Assessment

#### Executive Function

The computerized battery contained five cognitive processing tasks, including two response inhibition tasks (Go-No Go (GNG) and Anti-Saccade task (AS)), Task Switching (TS), a cognitive flexibility test, and two interference processing tasks (Flanker Shape (FS) and Shape Matching (SM), see Supplemental Table 7).

#### Go–No Go (GNG)

The mean correct hits in the GNG trials were 55%. Correct rejections were higher, at 89%. However, correct hits and rejections were as low as 4% and 9% respectively. There were nine youth who were considered poor performers at under 30% correct hits or rejections.

#### Anti-Saccade (AS)

The percentage correct for the anti-saccade task was 54% and the RT was 771 ms. There were 21 youth whose percentage correct was below 25% (chance) or had an RT above 1000 ms.

#### Task Switching

Mean TS scores were significantly higher during switching than the average reaction time (RT) of the non-switching tasks (*t*(98) = 18.220, *p* = 0.000). Mean Switch Cost (SC) was determined by subtracting the RT for switching from the average of RT for the non-switching conditions. Mean SC was 248 ms. Six participants were considered poor performers with a switch that cost longer than 1000 ms.

#### Flanker Shape

For the first interference processing task, the Flanker, RT were longer for interference and mixed trials than for control trials, and RT differed between control and interference trials (*t*(97) = 28.621, *p* = 0.000). There were nine participants who performed above the 750 ms cut-off point for the difference in RT between control and interference.

#### Shape Matching

On the second interference processing task, the Shape Match and control RT were faster than interference and mixed RTs, and the RT differed between control and interference (*t*(95) = 27.2348, *p* = 0.000). Seven participants had RTs greater than 500 ms, the poor performance cut-off. Thirty participants performed poorly on at least one indicator of executive performance.

#### Cognitive Assessments

##### Kaufman Brief Intelligence (KBIT-2)

The KBIT-2 has standardized scores between 85 and 115 to be considered a nationally normed average. Most participants scored toward the lower end of the average range for all categories of verbal (*M* = 91.69, *SD* = 14.03), nonverbal (*M* = 96.77, *SD* = 14.43), and IQ (*M* = 93.95, *SD* = 14.54), with a grade equivalent range of 7th to 8th grade. The minimum scores for all three measures are in the lower extreme range, which is a nationally normed score of 69 or below. A total of 11.3% of participants met verbal and/or non-verbal learning disability criteria (see Table 2).

**Table 2:** Results of Academic Testing

BRIEF-SR Mean T-Scores and count above a clinical score															
			N	Min	Max			*Mean*			*SD*		Clinical		
Inhibit T-Score			95	36	77			55.14			11.31		24 (25.0%)		
Shift T-Score			95	34	88			55.80			12.20		19 (19.8%)		
Emotional Control T-Score			95	38	85			57.43			11.24		23 (24.0%)		
Monitoring T-Score			95	37	84			51.97			11.08		16 (16.7%)		
Working Memory T-Score			95	36	84			56.43			12.39		11 (11.5%)		
Plan/Organize T-Score			95	34	80			49.81			10.76		9 (9.4%)		
Organization of Materials T-Score			95	36	75			47.82			8.85		7 (5.7%)		
Task Completion T-Score			95	37	75			52.66			10.42		15 (15.6%)		
Behavioral Regulation Index T-Score			95	34	81			56.43			11.02		28 (29.2%)		
Metacognition Index T-Score			95	34	82			50.81			10.64		10 (8.1%)		
Global Executive Composite T-Score			95	32	83			53.62			11.37		17 (13.8%)		
BRIEF-SR Clinical Scores		0		1				2		3					4+
N (%)		52 (54.2%)		12 (12.5%)				11 (8.9%)		11 (8.9%)					10 (10.3%)
Wisconsin Card Sorting Task Mean T-Scores and Count at least “mildly impaired”															
			N	Min	Max			*Mean*	*SD*					Clinical	
Total Errors			89	19	65			44.82	11.95					30 (33.7%)	
Perseverative Responses			89	20	72			46.67	11.29					27 (30.3%)	
Perseverative Errors			89	19	71			46.3	11.47					28 (31.5%)	
Conceptual Level Response			89	19	66			44.71	11.24					27 (30.3%)	
WCST Clinical Scores		0		1	2			3	4					5	
N (%)		53 (59.6%)		2 (2.2%)	5 (5.6%)			7 (7.9%)	5 (5.6%)					17 (19.1%)	
Kaufman Brief Intelligence Test (KBIT-2)															
			N	Min	Max	*Mean*			*SD*					Clinical	
Verbal Standard Score			88	62	130	91.69			14.03					9 (7.3%)	
Nonverbal Standard Score			88	48	130	96.77			14.43					4 (3.3%)	
IQ Standard Score			88	54	128	93.95			14.54					11 (12.5%)	
Wide Range Achievement Test (WRAT-3)															
Reading Standard Score			93	12	118	89.24			15.78					17 (19.8%)	
Math/Arithmetic Standard Score			96	45	120	84.53			15.27					26 (30.6%)	
Spelling Standard Score			95	49	123	90.23			15.99					15 (17.6%)	

Note: For the WCST T-scores 39 and lower are considered mildly impaired

##### Wide Range Achievement Test (WRAT-4)

Most participants scored slightly below average for all categories of the WRAT-3 (arithmetic (*M* = 84.53, *SD* = 15.27), reading (*M* = 89.24, *SD* = 15.78), and spelling (*M* = 90.23, *SD* = 15.99). Participants’ school grade equivalents for each skill were also slightly below average, with arithmetic being the lowest at about 7th grade (*M* = 6.85, *SD* = 3.28). Between 5.2% and 12.2% of youth scores were in the specific learning disability range.

##### Wisconsin Card Sort Task (WCST-CV)

Participant mean t-score on the WCST-CV were around 45, which is the low end of the average (45–54, see Heaton 1993, see Table 2). T-scores below 39 on this test are considered mildly impaired. 40% of youth had a T-score at the mildly impaired level or below in at least one of the five scores, with approximately 20% scoring at the mildly impaired level or below in all five main WCST indicators. Cronbach’s Alpha ranged from 0.75 to 0.94.

##### Behavior Rating Scale of Executive Function (BRIEF-SR)

The mean T-scores on the BRIEF-SR sub-scales were around 55, with a T-score above 65 considered clinically high. Approximately 25% of the population was in the clinical range for inhibiting and emotional control, with between five and twenty% of the population above clinical on the other measures. Only 54% of the population were not above a T-score of 65 on any sub-scale, with 27% above clinical on two or more sub-scales. Inconsistency and negativity scores were low, indicating that most participant responses were consistent and responded without an unusually negative manner (see Table 2). Cronbach’s Alpha ranged from 0.81 to 0.94.

### Disability Diagnosis

Using the three clinical measures (YSR, BDI, and TSCC), a total of 55.1% of the sample were above the clinical threshold for one mental health measure (see Table 3). Almost 20% of the participants were above clinical on one sub-scale of the YSR, BDI, or TSCC, and over 35% scored above clinical level on at least two sub-scales.

**Table 3:** Total Number of Mental Health Scales Above Clinical

	N (%)		N (%)
No Disability	44 (44.9%)	Clinical score on 4 scales	6 (6.1%)
Clinical score on 1 scale	19 (19.4%)	Clinical score on 5 scales	2 (2.0%)
Clinical score on 2 scales	13 (13.3%)	Clinical score on 6 scales	1 (1.0%)
Clinical score on 3 scales	11 (11.2%)	Clinical score on 8 scales	2 (2.0%)

Executive function (EF) tasks were included in the study to examine current cognitive functioning on tests of response inhibition, interference processing, and cognitive flexibility. Chi- square tests were used to examine if poor performance on EF would be more common in participants diagnosed with having a disability using the limited measures used in the present study. Mental Health disability, as assessed by the YSR, BDI, and TSCC, was not associated with poor performance on EF (*χ*^2^(1) > = 0.137, *p* = 0.71).

Using only eight academic measures, a total of 77.6% of participants had at least one disability diagnosis (see Table 4), and between 1.2% and 10.6% of youth were found to have a super-clinical score within one domain of functioning. 22.4% had super-clinical scores on two scales and between 1.2 and 14.1% scored above the clinical level on measures within more than three domains. EF score and academic disability were not associated (*χ*^2^(1) > = 1.95, *p* = 0.16).

**Table 4:** Total Number of Academic Disabilities Diagnosed with Individual Testing

	N (%)		N (%)
No Disability	19 (22.4%)	Clinical score on 2 scales	19 (22.4%)
Only Executive Function (BREIF-SR)	7 (8.2%)	Clinical score on 3 scales	12 (14.1%)
Only Frontal Lobe (WCST-CV4)	9 (10.6%)	Clinical score on 4 scales	5 (5.9%)
Only Specific Learning, Reading	4 (4.7%)	Clinical score on 5 scales	1 (1.2%)
Only Specific Learning, Math	3 (3.5%)		
Only Specific Learning, Spelling	2 (2.4%)		
Only Verbal	1 (1.2%)		
Only Non-verbal	1 (1.2%)		
Only Cognitive Delay	2 (2.4%)		

When the super-clinical counts were combined across mental health and academic functioning, nearly 85% of the participants were over clinical on at least one measure (see Table 5). 14.2% of the participants scored above the clinical cut-off on only one measure, while the other 60% scored above clinical by one, two, or more scales. There was no association between poor EF score and co-morbid disability status (*χ*^2^(1) > = 1.53, *p* = 0.2).

**Table 5:** Total Number of Academic Disabilities Diagnosed with Individual Testing Combined with Mental Health Above Clinical

	N (%)		N (%)
No Disability	14 (17.9%)	Clinical score on 2 scales	17 (21.8%)
Only Mental Health Subscale	2 (2.6%)	Clinical score on 3 scales	10 (8.1%)
Only Frontal Lobe (WCST-CV4)	3 (3.8%)	Clinical score on 4 scales	7 (5.9%)
Only Specific Learning, Reading	2 (2.6%)	Clinical score on 5 or more scales	19 (24.4%)
Only Specific Learning, Math	2 (2.6%)		
Only Verbal	1 (1.3%)		
Only Cognitive Delay	1 (1.3%)		

Finally, the overlap between disability and VE was examined using the *χ*^2^. Academic disabilities were more common in VE youth (*χ*^2^(1) > = 6.102, *p* = 0.014) but not mental health disabilities (*χ*^2^(1) > = 2.977, *p* = 0.084).

## Discussion

Many studies of adults who are incarcerated or former offenders have found that childhood VE (Baglivio et al. 2014; Ball 2005b; Casey et al. 2025; Fox et al. 2015; Jones et al. 2021a, b) and both academic and mental health disabilities are common. However, there are fewer studies that examine either of these issues in incarcerated youth (Astridge et al., 2023) and even fewer that investigate VE and disabilities in the same youth sample, particularly with direct testing of current functioning. The current study was conducted to fill this gap and test youth who are incarcerated for cognitive, academic, mental health functioning, and VE. This represents an opportunity to understand the challenges faced by this population, which is important for program development.

High levels of childhood violence exposure, childhood trauma, and disabilities were reported by both the youth self-report and records search. Overall, nearly the whole population was diagnosed with an academic/cognitive or mental health disability (85%) using both individual testing and well-regarded clinical self-report measures. These high disability rates were primarily driven by cognitive and academic disabilities, which we found is approximately 75% of the youth. Youth with severe VE were more likely to have academic disabilities, but not mental health disabilities. Although it is not possible with the current study to understand direction of effect, prior work has shown that academic and cognitive disabilities are a risk factor for childhood abuse and neglect (Author reference, 2012).

The participants reported violence exposure at high rates, with about three-quarters reporting at least some trauma exposure, two-thirds qualifying for a high ACEs score, and the average experiencing family violence at least a couple of times a year. This combination of high exposure to prior violence and a high proportion of youth with a diagnosable disability suggests that youth who are incarcerated come into the system with significant, but treatable, academic and mental health conditions. Knowledge of youth challenges upon entry into the juvenile justice system can help to develop targeted interventions for this population that can optimize the potential for reducing recidivism (Hall, 2015; Lockwood et al., 2012).

### Violence Exposure

Youth reported on average close to five previous experiences of ACEs, where four or more is generally considered a high ACEs score. Approximately two-thirds of these incarcerated youth reported at least four ACEs which is in line with findings of other researchers (Baglivio et al., 2014) and is well above the average 18.5% of youth who experience four or more ACEs (Swedo et al., 2024). A recent systematic analysis found that between 11 and 72% of incarcerated youth report ACEs and that the pooled estimate for this population is approximately 40%, similar to our findings (Astridge et al., 2023). Another systematic meta-analysis has reported that household dysfunction ranges from 10 to 36% of incarcerated male youth (Umpunjun et al., 2024) and between 12 and 38% on measures of child abuse and neglect.

Prior victimization was common according to other measures of violence exposure. The average youth reported at least a couple of times a year on the CTS exposure to interpersonal, parental, and sibling violence, while reporting that they themselves were involved in family violence at least a couple of times a year. An early study of formerly incarcerated adults found that nearly 90% experienced at least mild physical abuse in childhood (Ball, 2005a), which is similar to our findings in youth. In a study of incarcerated adult women, Jones and colleagues found a similar rate of experiencing IPV using the CTS; however, since this was an adult population, this is partner victimization, not childhood exposure to IPV (Jones et al. 2021a, b). Another Jones study of adult women found that perpetration of IPV was reported at a similar level (a few times a year) by both native and non-native women (Jones et al. 2021a, b).

Reports of childhood trauma according to the CTQ were very high. Close to 75% of youth reported at least a low level of childhood trauma on one childhood maltreatment sub-scale of the CTQ. A minimum of 25% of youth reported a severe level of CM on each of the five measures, with over 40% reporting severe physical abuse and a third reporting severe sexual abuse. These rates are similar to the prevalence rates found by Umpunjun (2024) in their study of incarcerated males. A study from Australia found that 17% of male youth met criteria for at least subthreshold PTSD using the CTQ; however, they reported much lower prevalence rates of severe scores in all five subscales (Moore et al., 2013). Recent studies of youth in Switzerland (Heller et al., 2022) and China (Zhao, 2021) also reported lower rates of childhood trauma. However, a Malaysian (Ahmad & Mazlan, 2014) and a separate Australian study (Papalia et al., 2022) also found similar rates to ours with 52% of males reporting moderate to severe SA in the Malaysian study and 77% reporting at least one form of maltreatment in the Australian study, suggesting some variation cross-nationally and across study procedures.

Finally, exposure to community violence (CV) is known to be associated with.

violent offending (Hartinger-Saunders et al., 2011) .Our population reported an average CV exposure of about once or twice a year according to the Richters and Martinez measure, which is higher than previous findings in at-risk youth (Thompson et al., 2007).

### Disabilities

Previous work has documented a link between disabilities and violent victimization in the general population (Author reference, 2012; Author reference, 2012) and a link in both incarcerated youth (Papalia et al., 2022) and adults (Jones et al. 2021a, b). Although the current study cannot test causal direction, self-reported and record-reported disabilities prior to incarceration suggest that, regardless of causal direction, the combination of disabilities and childhood violence exposure is a major risk factor for youth offending behavior. In fact, given that over 60% of the population had a prior IEP or diagnosed disability via records search, this suggests that disability is a major risk factor for later youth offending behavior.

This study found high levels of mental health, cognitive, and physical disabilities of all kinds, both in the records review of pre-incarceration diagnosis and in the studied assessments. While other research found that disability doubles the risk of later incarceration (Mallett et al., 2023), the present study found that 60% of the youth had an IEP and/or diagnosed disability in their medical or educational records and found high levels of current cognitive deficits. During cognitive testing, we found rates of verbal, non-verbal, spelling, reading, and math deficits between 3.5 and 30.6% of the population. This is much higher than expected in the general populations (Li et al., 2023), where studies found US rates of intellectual disabilities (ID) are around 1% and learning disabilities are 7%. This is also higher than rates found in a large study in Canada, where developmental disabilities were more than double in an adult prison population, but lower than the present study (Whittingham et al., 2020).

Disabilities spanned cognitive and emotional domains. Learning and cognitive disabilities are well-documented in incarcerated populations and are considered a risk factor for offending. Bixby (2022) found that 60% of adults in federal prisons had non-psychiatric disabilities, similar to our pre-incarceration rate of IEPs, and the rate of concurrent non-psychiatric clinical level scores we found in our population. This study is fairly unique in reporting individual cognitive and academic testing in an incarcerated youth population, and we were able to confirm through our own testing that the youth had high levels of cognitive disabilities. Just over 10% of the population scored below the cognitive delay cut-off on the KBIT-2, which is far higher than the national average (Li et al., 2023). Similarly, the population exhibited prevalence rates of learning disabilities that are far higher than national averages at 17–30% of the population. Nearly 45% of the population tested above the clinical threshold for executive functioning, as measured by the BRIEF-SR, and just over 40% tested at a level considered at least minimally impaired on the WCST, an indication of potential cognitive injury (Heaton et al., 1993). This rate far exceeds the percentage of adults with impairment, which was 14.6% in the original normal study.

Impaired scores on the WCST are known to be common in brain-injured populations, suggesting that undiagnosed brain injury may be common in this population. Our educational records search found that approximately 4% of our population had an IEP based on neurological disability, which suggests that at least some had a history of traumatic brain injuries (TBI). This rate is higher for neurological problems in youth (Banerjee et al., 2009). Although the present study did not have a direct measure of brain injury, a study of Australian detainees found that 33% reported head injury. Davies and colleagues (2012) found that more than 70% of their participant pool of incarcerated youth had experienced at least one head injury, and 41% experienced some loss of consciousness, indicating that TBI may be a common correlate with violent offending, as either cause or effect. Our findings from the WCST are consistent with this range of one to two-thirds of incarcerated populations showing signs of TBI.

Prior neurological injury may be a risk factor for detention for youth (Miura et al., 2005). Few studies have examined neuro-psychological function in incarcerated youth or adults, but some have found that impaired scores on the WCST are associated with anti-social and aggressive behaviors (Delfin et al., 2018). In a recent study, longer incarceration correlated with lower executive functioning and high impulsivity (Bauman et al., 2023), suggesting that lower EF is a risk factor for more serious offending. Our study indicates a pervasive problem with cognitive disabilities in youth who are incarcerated, including high rates of seizure activity and impairment in executive functioning, a finding that was supported by our cognitive functioning battery. With a 40% rate of clinical executive functioning deficit, this is consistent with prior work that shows that juvenile offenders had high impulsivity, more attentional bias, and lower executive function in various cognitive tests (Patiz & Bayraktar, 2023). The combined medical record search and mental health testing in our study support the findings that impulsivity is a problem for this population, with 28% having a previous diagnosis of ADHD and approximately 20% showing at least borderline clinical levels of attention problems with the YSR.

There was a very high rate of mental health diagnoses in the population as well. Approximately 20% of the population had moderate or severe depression according to the BDI-II. This is smaller than a recent study which showed that 25% had moderate depression, and 22% had severe depression (Domalanta et al., 2003). However, our study found that close to 45% of the population had at least one category where they scored above clinical on the YSR. Our sub-scale rates are compared to a recent study from Switzerland that found that incarcerated youth have a prevalence rate of 26.1% for externalizing disorders (ADHD, conduct disorders, and opposition disorders), and a prevalence rate of 18.8% for internalizing disorders (depression and anxiety; Heller et al., 2022). Our results are slightly higher than a recent Australian study that used the YSR and found rates of mean prevalence rates on the YSR between 4 and 16%, whereas our sub-scale clinical prevalence rates are between 10 and 27%.

Comorbidity of diagnosis was common with around 80% of the youth having at least one clinical level score on a diagnostic instrument. This is consistent with prior work from the last century documenting at least one diagnosis in 72% of youth in a small study of incarcerated youth (Atkins et al., 1999) and in two more studies: one of youth in temporary detention where the rate for males was 66% (Teplin et al., 2002), and one of incarcerated youth with an 83% rate of at least one psychiatric diagnosis (Heller et al., 2022). A more recent study from Canada found over a 90% rate of at least one diagnosis in incarcerated youth (Gretton & Clift, 2011), and one from the U.S. found a rate of 70% in incarcerated adults in federal prison (Bixby et al., 2022) with our findings adding to this body of work.

Our work suggests that comorbid diagnosis is predominantly driven by high rates of cognitive disabilities. Only 22% of the population did not fit criteria for diagnosis with an academic disability using the KBIT-2, WRAT-4, BRIEF-SR, and WSCT-CV. The largest group of youth who reported only one diagnosis exhibited either executive function or frontal lobe deficits (according to the BRIEF-SR and WCST-CV, respectively).

Approximately 35% of the youth exhibit a diagnosable level of academic functioning on two or three measures. This is higher than the Heller study (Heller et al., 2022) that found over 20% of youth with two or three comorbid diagnoses. More than 40% of the whole population showed evidence that they could be diagnosed with a minimum of two comorbid academic conditions, which is over 50% of the youth with a diagnosable academic condition. When mental health comorbidity is added, over 60% of the population was found to have more than one clinical level score. Few other studies have examined comorbidity in incarcerated youth, particularly when individual testing is conducted.

Incarcerated youth have significantly different health profiles than those of the same age. According to Forrest et al. (2000), incarcerated youth had significantly worse health compared to their counterparts in the domains of self-esteem, physical discomfort, acute disorders, chronic disorders, and psychosocial disorders. The worst health profile described 69.8% of the sample of incarcerated youth compared to the 30% in an age-matched school sample (Forrest et al., 2000). Only 6.4% of the incarcerated youth were in the good/excellent health profile, which was 34.2% of the school sample. Our own study showed particularly high rates of seizures, asthma, and bed wetting among individuals. A positive note from this study is relatively high self-esteem and strong attachment. This suggests that incarcerated youth have the basic skills necessary to harness the neural plasticity of youth.

Prior theoretical work on the social causes of juvenile delinquency suggests that violent victimization, including VE, is a major factor that is creating general strain (Broidy, 2001) as a cause of violent perpetration. Although our own work does not test the direction of effect, future research should focus on the role that comorbid disability and VE in predicting juvenile delinquency. Recent General Strain theory work highlights the role of teacher punishment (Moon et al., 2008) and bullying (Cullen et al., 2008) as particular social strains that predict perpetration. Both teacher punishment (MacSuga-Gage et al., 2021) and bullying (Bear et al., 2015) are more common in youth with disabilities, suggesting that juvenile justice theories may need to focus greater attention on role that prior cognitive and academic challenges play in juvenile perpetration (Mueller et al., 2019).

One limitation of the work is that this was a population of convenience that happened to be incarcerated at the time of the study, and missingness was not random. There may have been some patterns for youth who were released during the study which affected the missingness. As with all studies, findings should be replicated.

### Policy and Clinical Implications

One important finding from the current study is that youth who are incarcerated have high rates of academic and psychiatric diagnoses and VE; however, the direction of effect is unclear. Prevention of violent offending cannot always be attained; however, when youth are convicted, this is a potential inflection point for intervention. Strong academic, cognitive, and trauma-informed clinical interventions are necessary for the growth and development of this population while in the care of the state. As a policy, academic growth in youth incarceration facilities should be closely tracked. One implication of the research is that youth entering detention should be tested for cognitive impairments of various kinds. Youth who have not finished their education are often required to be involved in substantive education during their time of incarceration, which presents an opportunity to implement trauma-informed care and educational programming. One such program is an adaptation of The Heart of Learning and Teaching: Compassion, Resiliency, and Academic Success (HLT) curriculum (Wolpow et al., 2009), which has been successfully implemented in a youth facility for girls (Crosby et al., 2019).

Given that this population is known to be academically behind, large gains in academic functioning should be observed with strong intervention. There is work that shows that educational improvements decrease offending behavior, and this is an excellent time for targeted educational interventions.

### Social Justice Implications

Incarcerated youth are a special population that is understudied, and often, due to constraints on working in youth incarceration facilities, there are even fewer studies that obtain data from multiple sources. Inclusion of youth from facilities of all times is important for broader inclusivity in research. Educational and cognitive functioning data is particularly important for youth who are incarcerated. Because youth are in a position where they are often required to work toward a degree, understanding the need of this population and developing trauma-informed educational programs that target this population is necessary (see Herrenkohl et al., 2019) for a review of programs that have been implemented in public schools.

Through considering the participants’ experiences with adversity and exposure to violence, this study was able to identify cognitive disparities through the lens of social justice and inclusivity. Using medical records, surveys, and cognitive testing, we were able to recognize the health disparities in this population and the need for continued inclusive research within this population. Trauma-informed programs implemented in incarceration facilities have positive outcomes for mental health (Coelho et al., 2025), however there is little to no research on cognitive outcomes nor implementation in youth facility-based schools. The results of this study inform solutions to health equity in the future and suggest the need for the broader adoption of trauma-informed educational programs in schools.

### Future Directions

Future research should examine subgroups of high violence and disabilities and their aggression and offending behavior. Of particular interest is head injury in this population. It would be interesting to examine the relation that the WCST clinical level of functioning has with violence exposure in this population. That may give an indication of whether prior violence exposure could be considered causal, or at least connected, to evidence of TBI.

Future research should examine the relation between performance on test of cognitive function (such as the WCST), violence history, and mental health outcomes, such as aggression. Previous work has shown that prior VE is associated with offending behavior; however, it is unclear if disability is a mitigating factor.

## Supplementary Information

Below is the link to the electronic supplementary material.

Supplementary Material 1 (DOCX 41.4 KB)

## Data Availability

Data is available through Perkins, S. C., Smith-Darden, J., & Graham-Bermann, S. Cognition and Disabilities in Incarcerated Male Youth. Ann Arbor, MI: Inter-university Consortium forPolitical and Social Research [distributor], 2023-07-12. https://doi.org/10.3886/E147341V1.

## Declarations

### AI Statement

During the preparation of this work the authors used Consensus.app to find literature they may have missed through academic search tools. After using this service, the authors reviewed the original papers and take full responsibility for the content of the publication.
