# Supplementary material for: The Comorbidity of Violence Exposure, Mental Health, Cognitive Functioning, and Disabilities in Incarcerated Youth
Source: J Child Adolesc Trauma. 2026 Aug 26;19(4):2971–87. doi: 10.1007/s40653-026-00960-w (PMC13737232; doi:10.1007/s40653-026-00960-w)
Supplement: Supplementary file 1 [file 40653_2026_960_MOESM1_ESM.docx]

**Supplemental Text**

**S1. Expanded Methods Record Analysis**

**Medical Records.** Health data was gathered from the medical reports of the participants. Medications and drug usage per profile were collected and analyzed to identify a diagnosis. Using medication data, possible diagnoses were calculated, and statistics were obtained.

**Educational Records.** Data for special education diagnoses were collected from the educational records of the participants. The records included whether the participants were diagnosed, whether the participants qualified for specialized needs, and diagnoses of disabilities ranging from cognitive to physical impairments.

**Criminal Records.** Intake records were reviewed and coded for most recent offense type and class.

**S2. Expanded Methods Relationship Questionnaires**

***Self-Esteem.*** The Coopersmith Self-Esteem Inventory for school (C-SEI) questionnaire was used to examine youth self-reported self-esteem. The participants were asked to mark as “Like Me” (2) or “Unlike Me” (1), and items were reverse-coded following the scoring manual. The 58-item measure included a 50-item total self-measure and four factors: School-Academic (8 items), General-Personal (26 items), Home-Parents (8 items), and Social-Peer (8 items). Additionally, 8 items were specifically included for the Lie scale. A higher score indicated higher self-esteem.

***Attachment Style.*** Relationship Questionnaire (RQ, Bartholomew & Horowitz, 1991)) assessed attachment style with four descriptions of attachment patterns (secure, preoccupied, fearful, and dismissing). Participants are asked to indicate how well each paragraph describes them on a 7-point Likert scale (1 = It does not describe me at all, 7 = It very much describes me).

Higher scores indicate greater agreement with attachment patterns.

***Relationship Scale Questionnaire.*** The attachment style was measured by a modified version of the Relationship Scales Questionnaire (RSQ, Griffin & Bartholomew, 1994)), consisting of 17 items contributing to secure, dismissing, fearful, and preoccupied attachment patterns. Participants were asked to indicate how well each one described themselves when interacting with others on a 5-point Likert scale (1 = not like me to 5 = like me). The model of self and the model of others were calculated as reflections of an individual’s view of themselves (they are worthy of love and care) and their view of others (others are trustworthy and reliable)(Griffin & Bartholomew, 1994).

**S3. Expanded Methods Executive Functioning**

***Go-No-Go Task*** Response time and accuracy were measured. There were 130 items. The task lasted approximately 5 minutes. Correct hits and rejections were calculated. Hits and rejections were used to examine poor performance. Percent of correct hits and rejections below chance (30%) were counted as an indicator of poor performance.

***Anti-Saccade*** In the AS Task, participants were presented with a fixation cross in the middle of the screen. They were then presented with a box in one of the four corners with the instruction to look in the opposite direction of this box, where an arrow appears (saccade away). Participants then press the arrow keys to indicate the direction of the arrow. The arrow was masked after 150 ms. Reaction time and accuracy were measured.

***Task Switching*** For the TS task, participants were presented with prompt cues on the top left and right of the screen for 1500 ms. These prompt cues corresponded to the specific keys on the keyboard. They were presented with letter and number pairs. In the first set of tasks, they were asked to respond to the letter while ignoring the number and indicate whether the letter was a consonant or a vowel with a keyboard response. The second set required the participant to respond to the number of the letter/number pairs with the prompt cues “odd” and “even” presented before the trial. The final portion of the task randomly alternated between number trials and letter trials. There were 48 trials, and the 3 tasks lasted approximately 10 minutes combined. Reaction time and accuracy were measured for all three portions of the trials.^34^ Alternating trial blocks are used to determine the switch cost or the increase in reaction time when the task sets are switching versus the reaction time for single task blocks (Rogers & Monsell, 1995).

***Flanker Shape*** Eight small circles were presented in a larger circle formation. The shapes were presented within the small circles. The shapes were also presented inside and around the larger circle formation. The subjects were instructed to respond only to the shapes within the small circles and to ignore the flanking distractor shapes. A total of 130 blocks were presented, lasting approximately 5 minutes. Reaction time and accuracy were measured.

***Shape Matching*** A white shape was presented on the left and a green shape was presented on the right. The white shape on the left was superimposed over a red shape, which the subject was told to ignore. The participant was instructed to indicate if the right and left shapes were the same. Reaction time and accuracy were measured (Friedman & Miyake, 2004).

**S4. Expanded Results Record Analysis**

**Medical Records.** Over 40% of the youth had previously diagnosed depression, over 40% were taking antipsychotic medications at intake, and nearly 30% entered the facility with a diagnosis of attention deficit hyperactivity disorder (ADHD). Other physical health problems were common, with nearly one quarter having a previous history of seizures (see Supplemental Table 1).

The highest count of medical diagnoses was depression, asthma, antipsychotics, and ADHD. Around half of the incarcerated youth were on medications associated with depression. ADHD, antipsychotics, asthma, and seizure medications were among the most common medications taken by the incarcerated youth.

**Criminal Records.** Participants had been convicted of violent, sexual, and property crimes (see Supplemental Table 3). From the intake records, we were able to obtain information on crimes. Most of the participants had one criminal violation (62.2%), but many had 2 (17.8%) or 3 violations (20%). Violations were coded by state law into classes. Class one is the highest offense within each category. Thirty-six percent of the population committed a class two offense. Approximately equal numbers committed offenses in classes 1 and 3 (25%), with eleven percent having only a class four offense. The worst offense was categorized by class and offense type (see Supplemental Table 3). To avoid very small groups, class/offense groupings with fewer than five participants were combined with the next group. The largest part of the sample was Class 2 and Class 3 sexual offenders.

**S5. Expanded Results Relationship Questionnaires**

***Self-Esteem.*** The mean scores for self-esteem were between 1.3 and 1.6 on a 0 to 2 scale, with higher scores representing higher self-esteem. The Lie scale had the highest mean score (m = 1.63) but had low internal reliability with an alpha of only .24.

***Attachment Style.*** Youth were most likely to endorse a secure attachment style with a mean score of 4.26 on a 1-7 scale and with the lowest mean rate of endorsing the dismissing attachment style (m = 3.06).

***Relationship Scale Questionnaire (RSQ).*** The RSQ indicated a higher mean for secure (M = 3.26) and dismissing (M = 3.24) attachment styles than the other two styles. The results showed a positive model of self and others, where the model of self-ranges from -7 to 9, with a mean of 1.28, and the model of others ranged from -10 to 12, with a mean of 1.34.

| **Supplemental Table 1** | | | | | |
| --- | --- | --- | --- | --- | --- |
| *Descriptive Statistics from Medical Reports* | | | | | |
|  |  | Yes | | No | |
|  | N Total | N | Percent | N | Percent |
| Mental Health |  |  |  |  |  |
| Depression | 110 | 49 | 44.5 | 61 | 55.5 |
| Anxiety | 107 | 4 | 3.7 | 103 | 96.3 |
| Mood | 103 | 3 | 2.9 | 100 | 97.1 |
| ADHD | 110 | 79 | 28.2 | 31 | 71.8 |
| Antipsychotics | 109 | 47 | 43.1 | 62 | 56.9 |
| Physical Health |  |  |  |  |  |
| BP | 108 | 5 | 4.6 | 103 | 95.4 |
| Seizures | 109 | 26 | 23.9 | 83 | 76.1 |
| Gastrointestinal | 108 | 5 | 4.1 | 103 | 83.7 |
| Allergies and Eczema | 107 | 4 | 3.3 | 103 | 83.7 |
| Asthma | 105 | 16 | 15.2 | 89 | 84.8 |
| Bed Wetting | 104 | 5 | 4.8 | 99 | 95.2 |
| Acne | 103 | 4 | 3.9 | 99 | 96.1 |
| Diabetes | 103 | 4 | 3.9 | 99 | 96.1 |
| Migraines | 85 | 3 | 3.5 | 82 | 96.5 |
| Sleep | 45 | 1 | 2.2 | 44 | 97.8 |

| **Supplemental Table 2** | | | | | |
| --- | --- | --- | --- | --- | --- |
| *Descriptive Statistics for Special Education Diagnoses* | | | | | |
|  | N Total | N | Percent | N | Percent |
|  |  | Yes | | No | |
| IEP | 106 | 74 | 60.2 | 32 | 26.0 |
| Diagnosed Disability | 102 | 77 | 62.6 | 25 | 20.3 |
|  |  | History or Present | | Unclear | |
| Cognitive | 103 | 12 | 9.8 | 91 | 74.0 |
| Neurological | 102 | 5 | 4.1 | 97 | 78.9 |
| ADHD | 108 | 51 | 41.5 | 57 | 46.3 |
| Emotional Disturbance | 103 | 50 | 40.7 | 53 | 43.1 |
| Fine Motor | 102 | 2 | 1.6 | 100 | 81.3 |
| Gross Motor | 102 | 2 | 1.6 | 100 | 81.3 |
| Speech | 103 | 13 | 10.6 | 90 | 73.2 |
| Communication | 102 | 10 | 8.1 | 92 | 74.8 |
| Vision | 104 | 58 | 47.2 | 46 | 37.4 |
| Hearing | 102 | 10 | 8.1 | 92 | 74.8 |

| **Supplemental Table 3** |  |  |
| --- | --- | --- |
| *Percent of Sample Criminal Offense Type and Class* | | |
|  | N | Percent |
| Violent class 1 or 2 | 11 | 8.9 |
| Violent class 3 | 13 | 14.6 |
| Violent class 4 | 8 | 9.0 |
| Sexual class 1 | 17 | 19.1 |
| Sexual class 2 or 3 | 32 | 36.0 |
| Property class 1 and 3 | 8 | 9.0 |
| Total | 89 | 100. |
| Note: Violent class 2 had only 3 participants, sexual class 3 had only 2 and property class 1 had only 1. | | |

| **Supplemental Table 4** | | |
| --- | --- | --- |
| *Sample Family and Demographic Characteristics* | | |
|  | N | Percent |
| Family of Origin | | |
| Two parents | 37 | 36.6 |
| Mom headed household | 39 | 38.6 |
| Dad headed household | 7 | 6.9 |
| Relative | 15 | 14.9 |
| Foster care | 3 | 3.0 |
| Race/Ethnicity | | |
| White | 34 | 39.5 |
| African American | 30 | 34.9 |
| Multiracial | 19 | 22.1 |
| Hispanic, Asian or other | 3 | 3.5 |
| Age | | |
| 13-14 | 2 | 1.8 |
| 15 | 5 | 4.5 |
| 16 | 23 | 20.9 |
| 17 | 33 | 30.0 |
| 18 | 31 | 28.2 |
| 19 | 10 | 9.1 |
| 20 | 6 | 5.5 |
| Last Grade Completed | | |
| 7^th^ grade | 3 | 2.8 |
| 8^th^ grade | 15 | 14.2 |
| 9^th^ grade | 10 | 9.4 |
| 10^th^ grade | 18 | 17.0 |
| 11^th^ grade | 28 | 26.4 |
| 12^th^ grade | 27 | 25.5 |
| 1 year college | 5 | 4.7 |

| **Supplemental Table 5**  *Percent of Sample Previous Placements and Treatment* | | | | |
| --- | --- | --- | --- | --- |
|  | Yes | | No | |
| Previous Placements | N | Percent | N | Percent |
| Foster care | 38 | 37.6 | 63 | 62.4 |
| Group home | 18 | 18.0 | 82 | 82.0 |
| Locked detention | 88 | 88.9 | 11 | 11.1 |
| Previous Treatments |  |  |  |  |
| Psychological treatment | 87 | 85.3 | 15 | 14.7 |
| Substance abuse treatment | 15 | 15 | 85 | 85.0 |

| **Supplemental Table 6**  *Self-Reported Behavioral and Mental Health* | | | | | | | | | | |  |
| --- | --- | --- | --- | --- | --- | --- | --- | --- | --- | --- | --- |
| Trauma Symptom Checklist for Children (TSCC) | | | | | | | | | | |  |
|  | N | Min | Max | Below Clinical | | | Above Clinical | | | Alpha | |
| Post Traumatic (T-score) | 103 | 32.13 | 82.39 | 96 (93.2%) | | | 7 (5.7%) | | | 0.80 | |
| Anxiety (T-score) | 103 | 36.57 | 83.83 | 96 (93.2%) | | | 7 (5.7%) | | | 0.82 | |
| Dissociation (T-score) | 103 | 32.52 | 76.19 | 95 (92.2%) | | | 8 (7.8%) | | | 0.73 | |
| Anger (T-score) | 103 | 33.38 | 75.46 | 96 (93.2%) | | | 7 (5.7%) | | | 0.85 | |
| Depression (T-score) | 103 | 34.16 | 84.44 | 94 (91.3%) | | | 9 (8.7%) | | | 0.76 | |
| Clinical Count |  | 0 | 1 | 2 | | 3 | | | 4 | |  |
|  |  | 82 (79.6%) | 12  (11.7%) | 4  (3.9%) | | 2  (7.0%) | | | 3  (2.4%) | |  |
| Youth Self Report | | | | | | | | | | |  |
|  | N | *Mean* | *Subclinical* | *Borderline Clinical* | | | *Above Clinical* | | | |  |
| Internalizing Behavior | 106 | .54 |  |  | | |  | | | |  |
| Withdrawn |  |  | 95 | 3 (2.8%) | | | 11 (10.6%) | | | |  |
| Somatic Complaints |  |  | 84 | 7 (6.6%) | | | 15 (14.5%) | | | |  |
| Anxious/Depressed |  |  | 84 | 4 (3.8%) | | | 16 (15.4%) | | | |  |
| Social Problems |  |  | 87 | 9 (8.5%) | | | 17 (16.0%) | | | |  |
| Thought Problems |  |  | 93 | 8 (7.5%) | | | 11 (10.4%) | | | |  |
| Attention Problems |  |  | 88 | 4 (3.8%) | | | 16 (15.1%) | | | |  |
| Externalizing Behavior | 106 | .73 |  |  | | |  | | | |  |
| Delinquent Behavior |  |  | 75 | 18 (17.0%) | | | 29 (27.4%) | | | |  |
| Aggression |  |  | 87 | 8 (7.5%) | | | 17 (16.0%) | | | |  |
| Clinical Count | 0 | 1 | 2 | 3 | | 4 | | 5+ | | |  |
|  | 66 (57.4%) | 21  (18.3%) | 11  (9.6%) | 8  (7.0%) | | 4  (3.5%) | | 5  (4.3%) | | |  |
| Beck Depression Inventory | | | | | | | | | | |  |
|  | N | Mean | Minimal | *Mild* | *Moderate* | | | | *Severe* | |  |
|  | 106 | 0.57 | 64  (64.6 %) | 16 (16.2%) | 17  (17.2%) | | | | 2  (2.0%) | |  |
| Self-Reported Delinquency (SRD) | | | | | | | | | | |  |
|  | N | Min | Max | *Mean* | *SD* | | | | Alpha | |  |
| SRD mean total score | 98 | 0 | 48.22 | 6.95 | 8.65 | | | | 0.92 | |  |
| Violence mean | 98 | 0 | 31.11 | 3.26 | 4.88 | | | | 0.67 | |  |
| Theft mean | 102 | 0 | 70.46 | 7.22 | 12.85 | | | | 0.92 | |  |
| Public Disorder mean | 102 | 0 | 54.00 | 9.49 | 10.43 | | | | 0.76 | |  |
| Financial mean | 102 | 0 | 68.60 | 4.76 | 10.37 | | | | 0.64 | |  |
| Attachment Style | | | | | | | | | | |  |
| Secure | 97 | 1.00 | 4.80 | 3.26 | 0.79 | | | | 0.26 | |  |
| Preoccupied | 96 | 1.00 | 4.80 | 2.92 | 0.78 | | | | 0.51 | |  |
| Dismissing | 97 | 1.60 | 5.00 | 3.24 | 0.77 | | | | 0.54 | |  |
| Fearful | 98 | 1.00 | 5.00 | 2.85 | 0.97 | | | | 0.64 | |  |
| Self-Esteem Scale | | | | | | | | | | |  |
| Total Self | 96 | 1.08 | 1.75 | 1.36 | 0.15 | | | | 0.84 | |  |
| Social Self-Peers | 98 | 1.00 | 1.88 | 1.31 | 0.24 | | | | 0.64 | |  |
| Home-Parents | 97 | 1.00 | 2.00 | 1.39 | 0.27 | | | | 0.66 | |  |
| School-Academic | 98 | 1.00 | 1.88 | 1.39 | 0.23 | | | | 0.58 | |  |
| General-Personal Self | 98 | 1.00 | 1.73 | 1.35 | 0.16 | | | | 0.74 | |  |
| Lie Scale | 96 | 1.13 | 2.00 | 1.63 | 0.17 | | | | 0.24 | |  |

| **Supplemental Table 7**  *Descriptives for Executive Functioning Tasks* | | | | | | | | | | | | | | | | |
| --- | --- | --- | --- | --- | --- | --- | --- | --- | --- | --- | --- | --- | --- | --- | --- | --- |
| Response Inhibition Tasks | N | | | Min | | | Max | | | | *Mean* | | | *SD* | | |
| Go – No Go |  | | |  | | |  | | | |  | | |  | | |
| Correct Hits (Percent) | 100 | | | 0.04 | | | 1 | | | | 0.55 | | | 0.23 | | |
| Correct Rejections (Percent) | 93 | | | 0.32 | | | 1 | | | | 0.89 | | | 0.16 | | |
| Average of Correct Hits and Rejections | 89 | | | 0.09 | | | 0.96 | | | | 0.55 | | | 0.20 | | |
| Anti-Saccade |  | | |  | | |  | | | |  | | |  | | |
| Correct (Percent) | 78 | | | 0.06 | | | 0.98 | | | | 0.54 | | | 0.29 | | |
| Reaction Time (ms) | 78 | | | 306.33 | | | 1583.5 | | | | 711.32 | | | 312.35 | | |
| Task Switching |  | | |  | | |  | | | |  | | |  | | |
|  | | | |  | |  | | | |  | | |  | | |  |
| Consonant Vowel Reaction Time | | 96 | | | 257.29 | | | | 1300.00 | | | 669.98 | | | 236.18 | |
| Odd Even Reaction Time | | 96 | | | 208.00 | | | | 1351.00 | | | 693.85 | | | 273.07 | |
| Average Reaction Time | | 93 | | | 268.60 | | | | 1408.57 | | | 688.41 | | | 234.69 | |
| Task Switch Reaction Time | | 99 | | | 207.00 | | | | 2255.00 | | | 921.39 | | | 505.57 | |
| Switch Cost | | 93 | | | -819.72 | | | | 1588.48 | | | 248.41 | | | 464.21 | |
| Interference Processing | | | |  | |  | | | |  | | |  | | |  |
| Flanker Shape | | | |  | |  | | | |  | | |  | | |  |
| Control Reaction Time | | 98 | | | 276.00 | | | | 1512.29 | | | 780.33 | | | 269.90 | |
| Interference Reaction Time | | 99 | | | 218.75 | | | | 2399.12 | | | 1168.48 | | | 472.54 | |
| Mixed Trials Reaction Time | | 98 | | | 247.38 | | | | 1763.95 | | | 968.00 | | | 328.07 | |
| Difference in Reaction Time | | 98 | | | -1062.25 | | | | 948.14 | | | 350.31 | | | 323.69 | |
| Shape Matching | | | |  | |  | | | |  | | |  | | |  |
| Control Reaction Time | 97 | | 286.70 | | | | | 1600.90 | | | 899.42 | | | 320.49 | | |
| Interference Reaction Time | 97 | | 247.50 | | | | | 1986.00 | | | 1079.69 | | | 417.56 | | |
| Mixed Trials Reaction Time | 95 | | 313.172 | | | | | 1744.33 | | | 985.54 | | | 355.78 | | |
| Difference in Reaction Time | 97 | | -308.05 | | | | | 647.67 | | | 179.05 | | | 205.17 | | |
| Note: Reaction Time is in milliseconds (ms) | | | | | | | | | | | | | | | | |
